# Primary health care-level interventions targeting health literacy and their effect on weight loss: a systematic review

**DOI:** 10.1186/s40608-015-0035-7

**Published:** 2015-02-17

**Authors:** Nighat Faruqi, Catherine Spooner, Chandni Joshi, Jane Lloyd, Sarah Dennis, Nigel Stocks, Jane Taggart, Mark F Harris

**Affiliations:** Centre for Obesity Management and Prevention Research Excellence in Primary Health Care, UNSW Australia, Sydney, Australia; Centre for Primary Health Care and Equity, UNSW Australia, Sydney, Australia; Clinical and Rehabilitation Sciences, Faculty of Health Sciences, University of Sydney, Sydney, Australia; Discipline of General Practice, University of Adelaide, Adelaide, Australia

**Keywords:** Health literacy, Obesity, Systematic review, Intervention research, Primary health care

## Abstract

**Background:**

Enhancing individual’s health literacy for weight loss is important in addressing the increasing burden of chronic disease due to overweight and obesity. We conducted a systematic review and narrative synthesis to determine the effectiveness of lifestyle interventions aimed at improving adults’ knowledge and skills for weight loss in primary health care.

The literature search included English-language papers published between 1990 and 30 June 2013 reporting research conducted within Organisation for Economic Cooperation and Development member countries. Twelve electronic databases and five journals were searched and this was supplemented by hand searching. The study population included adults (≥18 years old) with a body mass index (BMI) ≥25 kg/m^2^ and without chronic disease at baseline. We included intervention studies with a minimum 6 month follow-up. Three reviewers independently extracted data and two reviewers independently assessed study quality by using predefined criteria. The main outcome was a change in measured weight and/or BMI over 6 or 12 months.

**Results:**

Thirteen intervention studies, all targeting diet, physical activity and behaviour change to improve individuals’ knowledge and/or skills for weight loss, were included with 2,089 participants. Most (9/13) of these studies were of a ‘weak’ quality. Seven studies provided training to the intervention deliverers. The majority of the studies (11/13) showed significant reduction in weight and/or BMI in at least one follow-up visit. There were no consistent associations in outcomes related to the mode of intervention delivery, the number or type of providers involved or the intensity of the intervention.

**Conclusions:**

There was evidence for the effectiveness of interventions that focussed on improving knowledge and skills (health literacy) for weight loss. However, there was insufficient evidence to determine relative effectiveness of individual interventions. The lack of studies measuring socio-economic status needs to be addressed in future research as the rates of obesity are high in disadvantaged population groups.

## Background

Globally, rates of overweight (defined as a body mass index [BMI] ≥ 25 kg/m^2^) and obesity (BMI ≥ 30 kg/m^2^) have been escalating in association with an increasingly sedentary lifestyle and especially with an increased energy intake [1,2]. Once considered a problem only in high-income countries, obesity is on the rise in low and middle income countries [3]. The high rates have contributed to the increased prevalence of chronic diseases such as cardiovascular disease (CVD), diabetes and cancers [2].

Health literacy is the degree to which individuals have the capacity (knowledge and skills) to obtain, process and understand basic health information and services needed to make appropriate health decisions [4]. Compared with people with adequate health literacy, those with inadequate health literacy have poorer understanding of their chronic diseases [5,6], physicians’ instructions [7] and health-related internet usage [8]. Low health literacy has been associated with more hospitalisations, greater use of emergency care, poorer adherence to medications, and is also more common among elderly persons with poorer overall health status and higher mortality rates [9]. Low health literacy has been associated with increased risk of CVD and diabetes in the Australian population [10]. Our own primary research has demonstrated an association between low health literacy and obesity among adults [11] although this has also been previously described in children [12].

Weight management comprises the primary prevention of excess weight gain, regain or loss and optimising health and reducing risk of disease (whether or not weight loss is achieved) [13]. People need strategies involving continuing lifestyle change, regular monitoring and support from primary health care (PHC) professionals who are considered to be the first line of intervention providers for weight management [14]. Multicomponent interventions targeting the three key lifestyle areas related to obesity – nutrition, physical activity (PA) and psychological approaches to behavioural change – are more likely to be effective in addressing overweight and obesity than single component interventions [14]. Patients need both knowledge and skills to engage in the lifestyle change involved in these complex interventions.

Von Wagner and colleagues’ review of health literacy [15] introduced a framework on the associations between health literacy and health outcomes. These are mediated by three principal domains of health actions (proposed by Paasche-Orlow and Wolf [16]), namely access and use of health care, patient-provider interactions, and self-care (management of health and illness). While acknowledging the importance of each of the three health action domains [15] in weight loss, our review focused on ‘management of health and illness’. Thus the main objective of this review was to evaluate the effectiveness of lifestyle interventions which aim to achieve weight loss by enhancing individual’s knowledge and/or skills for weight loss.

## Methods

### Study design

A systematic review with narrative synthesis.

### Inclusion criteria

Studies were eligible for inclusion if they met the criteria below (Table 1).

**Table 1:** **Study selection criteria**

**Publication language**	**English**
Publication date	January 1990 to June 2013
Place of study	OECD countries
Setting	PHC or PHC provider outside PHC setting or to individuals who were referred to the study by PHC professionals
Study type	An intervention study with a minimum 6 month follow-up period
Participants	Adults, aged ≥18 years BMI ≥25 kg/m^2^ No chronic disease
Intervention	A trial where the intervention aimed to achieve weight reduction through influencing the knowledge and/or skills of participants
Outcomes	Change in weight and/or BMI

#### Types of studies

Intervention studies (experimental or quasi-experimental trials with or without a control group) with a minimum six-month follow-up published in English between 1990 and end of June 2013 within the Organisation for Economic Cooperation and Development (OECD) member countries.

#### Setting

The intervention needed to be delivered in PHC, or by PHC professionals outside PHC, or to individuals who were referred to the study by PHC professionals. The definition of PHC used was:

*Socially appropriate, universally accessible, scientifically sound first level care provided by health services and systems with a suitably trained workforce comprised of multi-disciplinary teams supported by integrated referral systems in a way that: gives priority to those most in need and addresses health inequalities; maximises community and individual self-reliance, participation and control; and involves collaboration and partnership with other sectors to promote public health.* [17]

#### Types of participants

Men and women (≥18 years) with a BMI ≥ 25 kg/m^2^ at baseline and without chronic disease who were in ‘treatment’ for weight reduction.

#### Types of interventions

Interventions aiming to achieve weight reduction through changing diet and/or PA with or without psychological approaches to behaviour change by improving the participants’ knowledge and/or skills for weight loss.

#### Outcomes

Measured change in body weight or BMI (in kg/m^2^) between baseline and follow-up, at least once, at six months or beyond post intervention. For controlled studies these outcomes are compared between intervention and comparison groups. The outcomes were classified as statistically significant if the weight or BMI reduction reported was *p* < 0.05.

### Excluded studies

Studies were excluded if they included pregnant women or individuals diagnosed with CVD, diabetes, cancer or other chronic conditions or where pharmaceutical or surgical interventions were employed for weight loss.

### Search strategy

The electronic search covered the period from 1990 to June 2013. Twelve electronic databases (Medline, CINAHL, PsycINFO, APAIS-Health, Scopus, Embase, Cochrane Library, Web of Science, Australasian Medical Index, PAIS International, Joanna Briggs Institute Library, and Google Scholar) were searched using a comprehensive search strategy (Appendix 1). We complimented this with searching for references in five journals (*Patient Education and Counseling; American Journal of Preventive Medicine; Preventive Medicine; International Journal of Obesity;* and *Health Education & Behavior*). The selected studies were also used for identifying earlier and more recent publications.

Several relevant websites of key government, international bodies and non-government organisations were searched for grey literature. Experts in this area of research were also contacted for any relevant literature.

### Identification of relevant studies

CJ, JL and NF independently carried out initial screening of the retrieved titles and abstracts (where available) against agreed *a priori* criteria summarised in Table 1 (Step 1). A 10% random sample of excluded studies was reviewed by CS and JL (Step 2). Full-text copies of potentially eligible papers were obtained and independently assessed (Step 3) by seven reviewers (CJ, CS, JL, JT, NF, MH and SD). Data were independently extracted by three reviewers (CJ, MH and NF) into a summary table (Step 4). Any disagreements in specific study inclusion and/or data extraction were resolved through consensus by discussion.

### Quality assessment of studies

Quality assessment for each included study was carried out by CJ and SD using a standard checklist [18] and checked by NF. An overall methodological rating of strong, moderate or weak was achieved in six sections: 1) selection bias, 2) study design, 3) confounders, 4) blinding, 5) data collection methods, and 6) withdrawals and dropouts [18].

### Data extraction

Data were systematically extracted on the following domains:

*Study characteristics*: year of publication, design, recruitment method, location (country and setting), number of participants, study duration and length of follow-up (points of follow-up measurement), proportion of subjects lost to follow-up and appropriate control or ‘usual care’ group (where applicable).

*Intervention intensity:* Interventions were categorised into low, medium, high or very high intensity depending on the duration of contact between the provider and the participant or the number of points of contact between the two during the intervention period:

- low intensity: ≤4 hours of contact **or** 6 points of contact between the provider(s) and the participants;
- medium intensity: >4 hours and <8 hours of contact **or** 10 points of contact between the provider(s) and the participants;
- high intensity: ≥8 hours and <12 hours of contact **or** 12 points of contact between the provider(s) and the participants; and
- very high intensity: ≥12 hours of contact **or** 14 points of contact between the provider(s) and the participants.

*Participant characteristics*: baseline socio-demographic variables (gender, mean age, education, socio-economic status, employment), ethnicity, and risk factors.

*Intervention characteristics*: professional background of individuals delivering the intervention, mode of administration, component, dose of delivery (frequency and duration), and focus (knowledge, skills, behavioural change).

### Analysis

Change in measured weight and/or BMI was compared over 6 and/or 12 months. A meta-analysis could not be performed due to small number of studies identified and heterogeneity amongst the studies. A narrative synthesis approach was used.

## Results

### Trial identification

**Figure 1:**
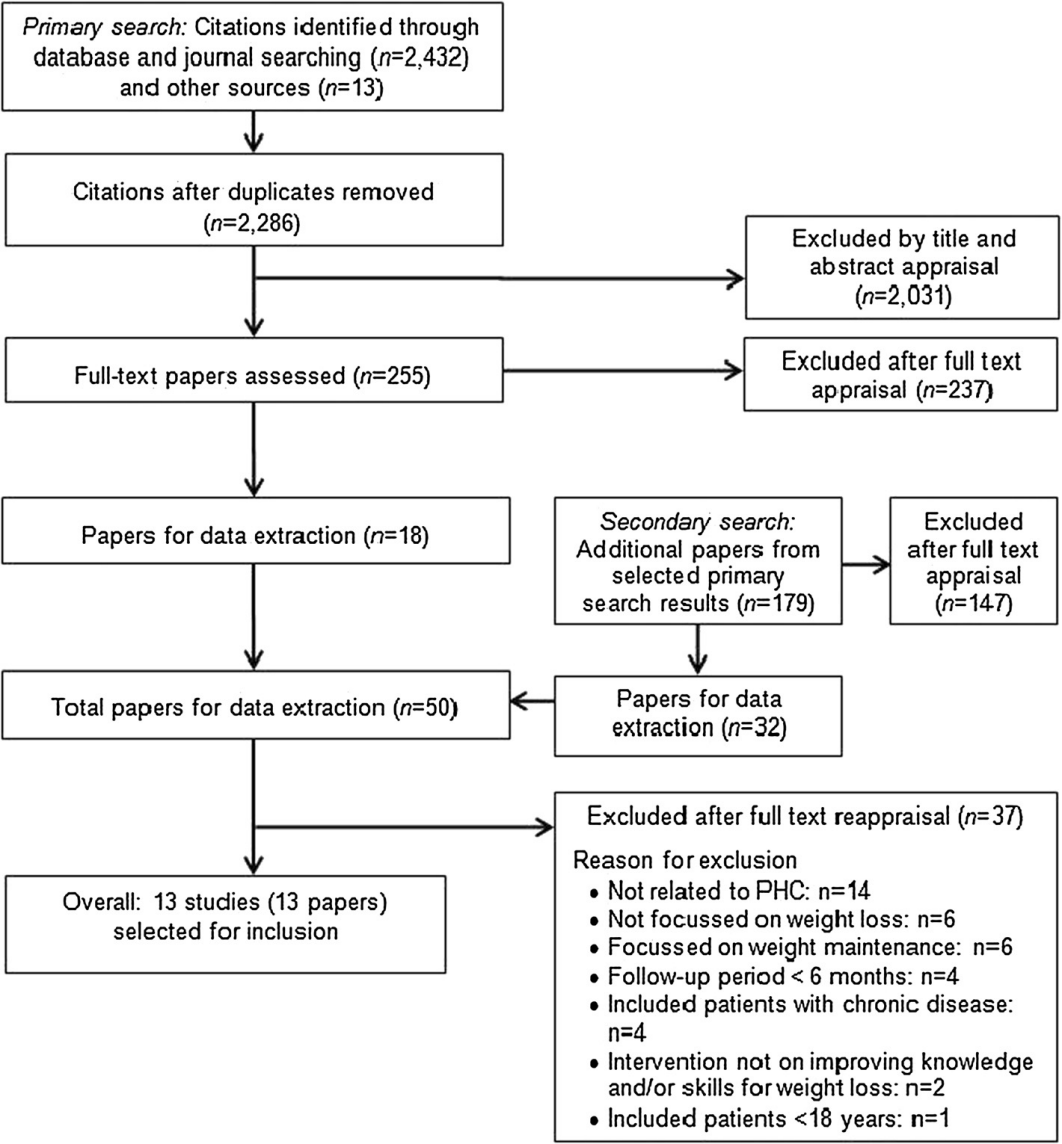
**Flow chart for study selection.**

The process of identifying and selecting papers for inclusion in this review is illustrated in Figure 1. Titles and abstracts (where available) of 2,286 papers were screened and 255 papers identified for full-text assessment. Of these, 18 papers were eligible for data extraction. From these, a further 179 papers were identified for a full text review. After excluding studies which did not meet the inclusion criteria, 13 papers were included in this review [19-31] (Figure 1).

### Study and participant characteristics

Seven of the included studies were randomised controlled trials (RCTs) [21,23,25,26,28,30,31], of which two were pilot studies [30,31] and one was a feasibility study using a delayed intervention control group [21]. Two studies had non-equivalent groups design [22,29] while four studies did not include a control group (pre-post single group designs) [19,20,24,27].

In almost all studies, patients were recruited from a PHC service, which was also the intervention setting. The exception was one study for which participants were recruited from community and the intervention was delivered in PHC [22].

The total number of participants across all studies was 2,089 (mean n = 161). Retention rates varied from 45 to 100% with eight studies retaining >80% of participants at the final follow-up.

From the methodological quality assessment, most of the studies were scored to be ‘weak’ in quality with only four studies scoring as ‘strong’ and none as ‘moderate’ (Table 2).

**Table 2:** **Quality assessment of studies**

**Study (Year)**	**Selection bias**	**Allocation bias**	**Confounders**	**Blinding**	**Data collection methods**	**Withdrawals and dropouts**	**Category of quality**
**Randomised controlled trials**							
Barclay (2008) [21]	Strong	Strong	Strong	Strong	Strong	Strong	Strong
Bo (2007) [23]	Strong	Strong	Strong	Strong	Strong	Strong	Strong
Greaves (2008) [25]	Strong	Strong	Strong	Strong	Strong	Moderate	Strong
Kulzer (2009) [26]	Weak	Strong	Strong	Weak	Strong	Strong	Weak
McConnon (2007) [28]	Weak	Strong	Strong	Weak	Strong	Weak	Weak
Tsai (2010) [30]	Weak	Strong	Weak	Weak	Strong	Strong	Weak
Whittemore (2009) [31]	Moderate	Strong	Strong	Strong	Strong	Strong	Strong
**Non-Equivalent Groups Design trials/ Single group pre-post trials**							
Absetz (2007) [19]	Moderate	Weak	Strong	Weak	Strong	Strong	Weak
Arrebola (2011) [20]	Weak	Weak	Strong	Weak	Strong	Weak	Weak
Bjorkelund (1991) [22]	Moderate	Weak	Weak	Weak	Strong	Strong	Weak
Gilis-Januszewska (2011) [24]	Weak	Weak	Weak	Weak	Strong	Moderate	Weak
Laatikainen (2007) [27]	Moderate	Weak	Strong	Weak	Strong	Moderate	Weak
Rohrer (2008) [29]	Moderate	Weak	Strong	Weak	Strong	Strong	Weak

Rating on study quality.

Strong: No weak and at least 4 strong ratings.

Moderate: 1 weak and <4 strong ratings.

Weak: ≥2 weak ratings.

### Intervention characteristics

The lifestyle interventions varied in the number of contacts with participants, mode of delivery, intervention providers, behaviour-change techniques (Table 3) and the duration of final follow-up. The modal period of intervention delivery was 6 months (n = 4), ranging from 3 to 12 months. The number of sessions ranged between five and 104 over the intervention period. The modal duration of final follow-up was 12 months (n = 8).

**Table 3:** **Details of the interventions**

**Study (Year)**	**Intervention period (months)**	**Sessions (number)**	**Duration of each session (hours)**	**Mode of administration and frequency**	**Intervention component**	**Intervention providers** ^**Ϫ**^	Intervention intensity score*
**Randomised controlled trials**							
Barclay 2008 [21]	6	6	1.5	Group sessions. First four held weekly, the fifth in week 12 and sixth in week 26	Nutrition education, PA sessions, group motivational discussions, completion of food diary and its analysis accompanied with brief written comments.	Nutritional scientist^@^, psychologist^@^, aerobics instructor^@^	3
Bo 2007 [23]	12	5	1	1-1 and group sessions	Group sessions sensitive to cultural differences and patient expectations. Individualised verbal and written recommendations and group sessions covering diet, PA and behaviour modification.	Nutritionists^@^, specialists in endocrinology^@^, internal medicine^@^	2
Greaves 2008 [25]	6	Up to 11	~0.5	1-1 (median 8) and telephone contacts (median 1.5)	Action plans made and assessed at subsequent contacts using relapse-management/relapse-prevention techniques and targets increased gradually to build/reinforce confidence over time. 1–1 motivational interviewing and diet and PA assessment, recommendations and target setting. Participants encouraged to self-monitor weight, PA and energy levels and to develop sustainable cognitive and behavioural skills for managing diet and PA.	Health promotion counsellors^&^	2/3
Kulzer 2009 [26]	4	12	~1.5	Group sessions. First eight, 1/w; last four, bimonthly	Intervention based on self-management theory. Verbal and written information on diabetes prevention and resources and worksheets on diet and PA.	Diabetes educators^#^, psychologists^#^	4
McConnon 2007 [28]	12	52	NR	Internet. Participants asked to log on to the intervention website at least once/w	Personalised and generic advice on diet and PA and behaviour therapy and tools and information to support dietary and PA behaviour change. Website designed to enable patients in self-management and to vary frequency of use according to own needs. Motivational statements generated.	Website^&^	4
Tsai 2010 [30]	6	8	1/4 -1/3	1-1. At weeks 0, 2, 4, 8, 12, 16, 20, and 24	Visits using handouts. Recommendations on dietary and PA behaviour, completion of food diary and review of food and PA records at each visit.	Medical assistants^&^	1/2
Whittemore 2009 [31]	6	11	NR	1-1 and telephone sessions	Culturally relevant education on nutrition, PA, and diabetes prevention, behavioural support in collaboratively identifying lifestyle change goals and problem-solving barriers to change, and motivational interviewing when participants were unable to achieve lifestyle goals.	Nurse practitioners^&^	2/3
**Non-Equivalent Groups Design trials**							
Bjorkelund 1991 [22]	3	12	Diet: 3 PA: 1	Group sessions. Separate diet and PA sessions once every 2^nd^ w	Food education, cooking activities and PA sessions.	Dietician^@^, physical training instructors^@^	4
Rohrer 2008 [29]	12	104	Group: 1.5 Phone and 1–1: NR	Weekly 1–1 and group sessions. Midweek telephone follow-ups	Behavioural classes focussing on lifestyle change and meal replacements including record keeping, goal setting, planning, problem solving, environmental control, and PA.	Nurses^@^, health educators^@^	4
**Single group pre-post trials**							
Absetz 2007 [19]	8	6	2	Group sessions. First five at 2w intervals, last at 8 m	A group-based, task-oriented counselling model base on the Health Action Process Approach (HAPA). The program emphasised the participants’ possibilities to make informed choices and his/her role as an independent decision-maker. Information provision, group discussions, behaviour self-monitoring, dietary counselling, diet and PA goal setting, planning, and motivation for life-style changes that would result in the diet and PA goals accomplishment.	Dietitian^^%^, public health nurses*, physiotherapist^$^, municipal sports officers^%^	4
Arrebola 2011 [20]	5.5	11	NR	One 1–1 and group sessions. Group sessions every 2w	Nutrition education, PA recommendations and psychological support.	Dietician ^~^^#^, nurse^#^, doctor^#^	2/3
Gilis-Januszewska 2011 [24]	10	18	NR	Group sessions, telephone, and letters. Intensive phase (4 m): 10 sessions. Continuous phase (6 m): 6 telephone sessions and 2 letters	Intervention based on reinforced behaviour modification. Social support emphasised by the group setting and participants encouraged to involve their own social environment in the lifestyle changes. Group sessions on lifestyle changes and diet and PA education using printed resources. PA sessions.	Nurses^&^	4
Laatikainen 2007 [27]	8	6	1.5	Group sessions. First five at 2w intervals, last session at 8 m	Intervention model used the HAPA. Regular self-assessment used to empower participants to take responsibility for own decisions and make informed choices. Social support enhanced by the group setting and encouraging participants to seek support from their own social networks. Goal setting used to motivate individuals to progress from intention to actual behaviour change. Education on diet and PA.	Dietitians^@^, nurses^@^, physiotherapists^@^	3

*Intervention intensity score.

1: Low - ≤4 hours of contact or 6 points of contact.

2: Medium - >4 hours and <8 hours of contact or 10 points of contact.

3: High - ≥8 hours and <12 hours or 12 points of contact.

4: Very high - ≥12 hours of contact or 14 points of contact.

^**Ϫ**^ **Provider.**

^^^: Supervised/supported the main intervention provider.

^#^: One or the other.

*: Main intervention provider.

^@^: All delivered.

^$^: Helped the main intervention provider.

^%^: delivered 1 session only.

&: Sole deliverer.

~: Solely delivered instructions on diet and PA during a 1–1 session.

A number of modes of intervention delivery were used: All the studies involved face-to-face contact, along with telephone and mail in some cases, except one that utilised the Internet and delivered the intervention through a website [28]. Of the 12 studies using face-to-face education, six used group sessions only [19,21,22,24,26,27], three used a combination of one-to-one and group sessions [20,23,29] and three individual contacts only [25,30,31].

The studies utilised a range of providers. There were six studies with one or one main deliverer of the intervention [19,24,25,28,30,31]. Of these, three studies used providers other than PHC professionals to promote weight reduction. One study evaluated an intervention delivered in the PHC setting by non-National Health Service staff (health promotion counsellors) [25], another used an Internet-based weight-control package in a community setting [28] and the third study, [30] evaluated the effectiveness of medical assistants as weight loss counsellors. Five studies had 2–3 intervention providers delivering the intervention [21-23,27,29] and in two studies, even though there were multiple intervention providers, only one led the group sessions [20,26].

Seven of the 13 studies provided training to health professionals and educators to deliver the intervention, however, only five provided details of the training [19,24,25,30,31], the duration of which ranged from three hours to nine sessions of six hours each. Two papers provided no details of the training [23,27].

Of the seven studies that included patients at risk of developing type 2 diabetes, three provided patients with information on diabetes prevention [21,26,31]. The one study where the participants had metabolic syndrome used a general recommendation-based program of lifestyle intervention carried out by trained professionals [23].

### Types of interventions

All the reviewed studies included interventions that, in combination, focussed on diet, PA and psychological approaches to health behaviour change. All studies explicitly stated that they targeted participants’ dietary knowledge except one where the emphasis was on meal replacement [29]. In six studies participants were provided educational resources/tools [19,23,24,26,28,30] and in one study participants attended dietitian-supervised cooking classes [22]. Participants were encouraged to keep food records [29], given diaries [19,26] and PA logbooks [26], provided with analysed nutritional data and brief comments on food diaries [21] or given the opportunity to review their completed food diaries and PA records with education deliverers [30].

In eight studies PA education [20,23-25,27,28,30,31] was given. One study also provided individualised advice on exercise [23]. In five studies participants could attend PA session(s) [19,21,22,24,29].

A lifestyle change personal goal setting approach was used by a number of studies to motivate participants to progress from intention to actual behaviour change [19,27-29,31]. Motivational interviewing [25,31], group motivational discussions [21], telephone motivation sessions along with motivation letters [24], and website-generated motivational statements [28] were used to modify participants’ behaviour and achieve weight loss. Participants were encouraged to self-monitor behaviour [19] and also develop cognitive and behavioural skills for managing diet and PA [25] or to self-manage weight [28]. In one study, [27] regular self-assessment was used to empower participants to take responsibility for own decisions and make informed choices. Other behaviour techniques included planning [25,29] and problem solving and environmental control [29].

The social support enhanced by the group setting in 9/13 studies mentioned earlier was further emphasised in two studies by encouraging participants to seek support from their own social networks [24,27].

### Weight loss

All 13 included studies measured change in body weight and 10 studies also measured change in BMI [19-24,26-28,31]. The study characteristics and outcomes are presented separately for RCTs (Table 4), non-equivalent group designs (Table 5) and pre-post designs without a control group (Table 6). Overall, in 11 of the 13 studies (85%) there were significant weight reductions as measured by weight or BMI [19-27,29,30]. Among these 11 studies, two did not show significant changes at all the follow-up periods [22,30] and a third study reported significant weight loss in the male but not female participants [19].

**Table 4:** **Summary of characteristics and results at 6 and 12 months for the included randomised controlled trials**

**Study (Year)**	**Participants**	**Interventions**	**Follow-up (months)**	Weight*/BMI**	**Intervention group**			**Control group**			**Between-group significance**
					**Pre mean**	**Post mean**	**Mean difference**	**Pre mean**	**Post mean**	**Mean difference**	
Barclay 2008 [21]	Location: UK Setting: PHC	Allocated: Immediate entry (Intervention group): 19 Delayed entry: 18 (of these, 11 formed the Control group)	6	Wt	85.5 (range 58.4–128.8)	NR	−2.73 (3.15)	85.8 (range 73.1–96.8)	NR	−0.30 (1.36)	p < 0.05
	Risk factor: T2DM	% dropout: 6.7		BMI	29.8 (range 23.1–43.0)	NR	−0.91 (1.01)	29.5 (range 22.8–35.5)	NR	0.10 (0.47)	p < 0.05
	Sex: (a) Immediate entry (Intervention group): women 13, men 6 (b) Delayed entry (Control group): women 4, men 7	Comparison: Diet-PA vs. Usual Care (D-PA vs. UC)									
	Age, mean (range): (a) 62.3 (50–83) (b) 67.5 (56–85)										
Bo 2007 [23]	Location: Italy Setting: PHC	Allocated: (a) 169 (b) 166	12	Wt	81.7 (14.9)	81.0 (15.7)	−0.75 (95% CI −1.49, −0.003)	81.3 (13.5)	82.9 (14.0)	1.63 (95% CI 0.83, 2.42)	p < 0.001
	Risk factor: Metabolic syndrome	% dropout: 0									
	Sex: (a) 99 women, 70 men (b) 96 women, 70 men	Comparison: D-PA vs. UC									
	Age, mean (SD): (a) 55.7 (5.7) (b) 55.7 (5.6)			BMI	29.7 (4.1)	29.4 (4.4)	−0.29 (95% CI −0.56, −0.02)	29.8 (4.6)	30.4 (4.8)	0.61 (95% CI 0.31, 0.91)	p < 0.001
Greaves 2008 [25]	Location: UK Setting: PHC	Allocated: (a) 72 (b) 69	6	Wt	91.6 (13.3)	91.3 (13.7)	NR	94.4 (14.2)	92.6 (15.0)	NR	p < 0.05
	Risk factor: T2DM	% dropout: (a) 19.4 (b) 17.39									
	Sex: (a) 46 women, 26 men (b) 44 women, 25 men	Comparison: D-PA vs. UC									
	Age, mean (SD): (a) 53.3 (12.3) (b) 54.5 (11.5)										
Kulzer 2009 [26]	Location: Germany Setting: PHC	Allocated: (a) 91 (b) 91	12	Wt	92.1 (16.5)	88.3 (15.9)	−3.8 (5.2)	93.6 (19.3)	92.2 (19.4)	−1.4 (4.0)	p = 0.001
	Risk factor: T2DM	% dropout: 9.3									
	Sex: 78 women, 104 men	Comparison: D-PA vs. written information									
	Age, mean (SD): 56.3 (10.1)			BMI	31.0 (4.7)	29.7 (4.7)	−1.3 (1.7)	32.0 (5.7)	31.5 (5.8)	−0.5 (1.4)	p = 0.002
McConnon 2007 [28]	Location: UK Setting: Community	Allocated: (a) 111 (b) 110	12	Wt^	98.9 (17.7)	93.6 (13.3)	−1.29 (5.6)	97.9 (17.1)	95.6 (17.7)	−1.9 (5.8)	NS
	Risk factor: Nil	% dropout: (a) 51 (b) 30									
	Sex: 170 women, 51 men	Comparison: Internet vs. UC									
	Age, mean: (a) 48.1 (b) 47.4			BMI^	36.1 (5.8)	34.9 (4.6)	−0.41 (2.0)	35.9 (5.1)	34.9 (5.0)	−0.7 (2.1)	NS
Tsai 2010 [30]	Location: USA Setting: PHC	Allocated: (a) 24 (b) 26	6	Wt	97.0 (SE 3.4)	NR	−4.4 (SE 0.6)	103.1 (SE 3.5)	NR	−0.9 (SE 0.6)	p < 0.0001
	Risk factor: Nil	% dropout: (a) 8.4 (b) 3.9									
	Sex: 44 women, 6 men	Comparison: brief behavioural counselling vs. no counselling	12	Wt	97.0 (SE 3.4)	NR	−2.3 (SE 0.9)	103.1 (SE 3.5)	NR	−1.1 (SE 0.8)	NS
	Age, mean (SE): (a) 51.3 (2.3) (b) 47.6 (2.5)										
Whittemore 2009 [31]	Location: USA Setting: PHC	Allocated: (a) 31 (b) 27	6	Wt, % loss	NR	NR	NR	NR	NR	NR	NS
	Risk factor: T2DM	% dropout: (a) 22.6 (b) 0		BMI	NR	NR	NR	NR	NR	NR	NS
	Sex: (a) 28 women, 3 men, (b) 24 women, 3 men	Comparison: Enhanced standard care vs lifestyle intervention									
	Age, mean (SD): (a) 48.2 (12.4) (b) 43.2 (13.2)										

Wt: Weight.

BMI: Body mass index.

*kg.

**kg/m^2^.

^^^Obtained by personal communication.

Mean (SD) unless specified otherwise.

**Table 5:** **Summary of characteristics and results at 6 and 12 months for the included non-equivalent groups design trials**

**Study (Year)**	**Participants**	**Interventions**	**Follow-up (months)**	Weight*/BMI**	**Intervention group**			**Control group**			**Between-group significance**
					**Pre mean**	**Post mean**	**Mean difference**	**Pre mean**	**Post mean**	**Mean difference**	
Bjorkelund 1991 [22]	Location: Sweden Setting: Community	Allocated: (a) 22 (b) 27 (c) 16	6	Wt							
				a.	88.1 (9.8)	NR	−3.98 (3.92)	87.3 (9.6)	NR	−1.77 (3.31)	p < 0.05
				b.	70.2 (7.7)	NR	−2.83 (2.46)	70.9 (7.6)	NR	−0.42 (2.55)	p < 0.001
				c.	90.6 (9.0)	NR	−2.87 (3.65)	88.8 (14.8)	NR	−1.41 (4.79)	NS
	Risk factor: Nil	% dropout (at 6 months): (a) 0 (b) 4 (c) 6		BMI							
				a.	33.0 (3.0)	NR	−1.50 (1.60)	33.2 (3.5)	NR	−0.65 (1.28)	p < 0.05
				b.	26.4 (1.8)	NR	−1.03 (0.95)	26.4 (2.5)	NR	−0.11 (0.96)	p < 0.001
				c.	33.9 (3.1)	NR	−1.04 (1.31)	33.4 (3.2)	NR	−0.51 (1.88)	NS
	Sex: all women	Comparison: Participants vs. non-participants of the intervention program, each in 3 groups: a. BMI ≥ 30 & W/H ratio < 0.82 b. BMI < 30 & W/H ratio ≥ 0.82 c. BMI ≥ 30 & W/H ratio ≥ 0.82									
	Age, range: 45-64										
Rohrer 2008 [29]	Location: USA Setting: PHC	Allocated: (a) 71 (b) 55	12	Wt^$^	NR	NR	−18.8 (15.7)	NR	NR	0.87 (5.9)	p < 0.001
	Risk factor: Nil	% dropout: (a) 18.4 (b) 18.2									
	Sex: (a) 50 women, 21 men (b) 38 women, 17 men	Comparison: Intensive structured vs. less intensive									
	Age, mean (SD): (a) 45.3 (11.6) (b) 48.9 (9.4)										

Wt: Weight.

BMI: Body mass index.

*kg.

**kg/m^2^.

Mean (SD).

^$^Not based on an ITT analysis.

**Table 6:** **Summary of characteristics and results at 6 and 12 months for the included pre post trials**

**Study (Year)**	**Participants**	**Interventions**	**Follow up (months)**	Weight*/BMI**	**Pre mean**	**Post mean**	**Mean difference**	**Within-group significance**
Absetz 2007 [19]	Location: Finland Setting: PHC	% dropout: 9.4	12	Wt Male Female	100.0 (18.1) 86.0 (13.2)	98.5 (18.1) 85.5 (13.3)	NR	p < 0.01 NS
	Risk factor: T2DM							
	Sex: 265 women, 87 men	Comparison: Before and after						
	Age, mean (SD): 58 (4.3) women, 59 (3.7) men			BMI Male Female	32.0 (5.3) 32.5 (4.6)	31.5 (5.2) 32.3 (4.7)	NR	p < 0.01 NS
Arrebola 2011 [20]	Location: Spain Setting: PHC	% dropout: 55	6	Wt	86.0 (15.6)	79.2 (13.4)	NR	p < 0.001
	Risk factor: Nil			BMI	32.0 (2.9)	29.4 (2.9)	NR	p < 0.001
	Sex: 43 women, 17 men	Comparison: Before and after						
	Age, mean (SD): 40 (9)							
Gilis-Januszewska 2011 [24]	Location: Poland Setting: PHC	% dropout: 0	12	Wt	85.6 (16.1)	83.7 (15.9)	1.9 (5.0)	p < 0.05
	Risk factor: T2DM							
	Sex: 137 women, 38 men	Comparison: Before and after						
	Age: NR			BMI	31.7 (5.0)	31.0 (4.9)	0.6 (1.9)	p < 0.05
Laatikainen 2007 [27]	Location: Australia Setting: PHC	% dropout: 23.8	12	Wt	91.7 (17.7)	NR	−2.5 (95% CI −3.19, −1.85)	p < 0.05
	Risk factor: T2DM							
	Sex: 172 women, 65 men	Comparison: Before and after						
	Age, mean (SD): 56.7 (8.7)			BMI	33.5 (5.9)	NR	−0.9 (95% CI −1.17, −0.69)	p < 0.05

Wt: Weight.

BMI: Body mass index.

*kg.

**kg/m^2^.

Mean (SD) unless specified otherwise.

Of the 11 studies with positive results, three were rated as ‘strong’ (all RCTs) [21,23,25] and the other eight were rated as ‘weak’ quality. In one of these studies (Greaves et al.) there was an increased proportion of patients achieving weight targets in the intervention but not control group [25]. However there was no significant change in mean weight in the intervention group and this group had lower initial weight compared to the control group (Table 4). Of the two studies that did not show significant reduction in either weight or BMI, one was rated as ‘weak’ [28] and the other as ‘strong’ quality [31].

There were no consistent differences in the effectiveness of interventions by their mode of delivery (except for the one Internet only intervention which was not effective), provider, behavioural intervention or intensity (Tables 4-6).

## Discussion

Our paper has reviewed publications reporting lifestyle interventions at the primary health care-level that aimed to increase adults’ knowledge and skills for weight loss. We identified only 13 studies fulfilling the inclusion criteria, the majority of which demonstrated a positive impact on weight loss. Only one included study reported outcome data for both 6 and 12 month periods [30]. Consistent with earlier systematic review evidence [32,33], this study showed weight regain after an initial weight loss which was, though, in keeping with the study hypothesis.

The studies included in our review were heterogeneous, the only commonality being the intervention focus, which in every case was to change diet and PA behaviours in conjunction with behaviour change. Included amongst the modes of delivery were individual and/or group sessions or the Internet with the number of sessions ranging from five to 104 conducted over 3 to 12 months. Educators were drawn from different professional backgrounds and included both health and non-health professionals. Only seven studies mentioned training the educators in the intervention delivery, suggesting a need for evaluated programs to more explicitly describe the training provided to the intervention deliverers.

This review reinforces earlier systematic reviews’ findings which support the efficacy of combining both dietary and PA interventions together with behaviour modification [34,35]. However, these earlier reviews did not necessarily include only those lifestyle interventions which specifically aimed to impact individual’s knowledge and skills required for weight loss. Despite the diversity in the types of interventions included in this review, the results provide evidence to support the role of lifestyle interventions aiming to change individual’s knowledge and/or skills in weight loss. Eleven out of 13 studies reported positive intervention effects. In the small number of studies identified, no one type or component of lifestyle intervention emerged as the most effective model and we were thus unable to determine what constituted the successful aspect of the intervention. By the same token, our review could not ascertain why interventions in two studies [28,31] failed to accomplish their objectives.

Reviews on the relationship between health literacy level and health outcomes have shown a consistent association between low health literacy and poorer health-related knowledge and comprehension [9,36]. None of the studies included in this review reported on participants’ health literacy. There is thus a need to address this gap in research and to develop weight loss interventions which specifically target people’s health literacy. Similarly, none of the included studies had specifically targeted or measured outcomes in disadvantaged socio-economic population. This is despite obesity being particularly prevalent among those in the most disadvantaged socio-economic groups [14] and disadvantaged populations struggling most with limited health literacy [37]. Future research is needed to evaluate interventions with this oft-neglected population group. Other approaches which develop and draw upon the health literacy located in wider community networks may need to be considered in future research with socially disadvantaged groups [38].

### Strengths and limitations

The included studies were based in a large number of countries with different health systems, obesity issues and population characteristics including Australia, the UK, the USA and European countries. The review is limited, however, by the small number of studies that met our selection criteria and were of high quality. With this limitation, it was not only difficult to confidently identify the effectiveness of the weight loss interventions, it was also not possible to identify individual intervention components associated with success.

Our search was not limited to studies that had tested participants’ health literacy for weight loss at baseline so we cannot ascertain if participants had low health literacy at the start. In addition, the included studies did not explicitly measure improvements in health literacy for weight loss. Though the vast majority of studies did lead to weight loss, we are unable to report specifically on health literacy improvements.

Most of the studies did not state the participants’ ethnicity, education and socio-economic status. This limits our capacity to understand the generalisability of our findings to people with different socio-economic status and ethnic backgrounds. Obesity often coexists with other chronic conditions; however, our review excluded patients with existing chronic diseases thus limiting the scope of the review findings to people who are in otherwise good health.

The heterogeneity of the studies precluded us from performing a meta-analysis. Furthermore, the review was not limited to RCTs. On the one hand, this allowed us to examine the efficacy of interventions in less controlled situations, while on the other hand studies displaying biases were also included. Most of the studies did not report on the reasons for participant dropout and more than half of the studies did not conduct the analysis on an intention-to-treat basis.

We included studies where the minimum follow-up period was six months and used outcome data only for 6 and 12 months. However, weight loss achieved in the first 6 months after intervention is often regained in the subsequent months as demonstrated by one of the reviewed studies [30]. Thus for studies where the final follow-up was at six months, we cannot exclude the possibility of weight regain over longer follow-up periods.

## Conclusions

Improving health literacy for weight loss in a PHC setting is a complex task and difficult to achieve. Health literacy in weight loss requires not only an understanding of what is required to lose weight but also an insight into the factors that prevent individuals from weight loss and promote weight regain. As our adopted framework demonstrates, these are important prerequisites for building motivation for change and the ability to achieve health goals.

Some promising results were found for complex multicomponent lifestyle interventions conducted at the PHC-level that focussed on weight loss. However, there was insufficient evidence to discriminate between the effectiveness of intervention components. Interventions of at least medium intensity delivered by a range of health professionals, which addressed both diet and PA and using behavioural strategies, were effective. More research is needed to explore the pathway between the intervention components, health literacy, behaviour, weight loss and, in the longer term, maintenance of these losses. The lack of studies measuring socio-economic status needs to be addressed in future.

## Appendix 1

### Electronic databases

#### 1. *Medline:* 221 search results

Health literacy/Literacy:

Health literacy/or patient education as topic/or “physical education and training”/Educational Status/or Health Education/Health Knowledge, Attitudes, Practice/or Attitude to Health/Patient Compliance/interactive health literacy.tw./critical health literacy.tw./(functional adj health adj literacy).tw.

Population:

Cultural deprivation/Homeless Persons/or Homeless Youth/Vulnerable Populations/Alcoholics/Drug Users/Prisoners/Refugees/“Transients and Migrants”/asylum seek*.mp. or “Emigrants and Immigrants”/Oceanic Ancestry Group/or Indians, South American/Inuits/(aboriginal and torres strait island*).mp./socioeconomic factors/ or poverty/

Risk factors:

Feeding behavior/or habits/or health behavior/exp Exercise/exp Obesity/or exp verweight/or exp Body Weight/exp Life Style/exp Diet/nutrition disorders/or overnutrition/

Primary Health Care:

Patient care management/or comprehensive health care/or primary health care/or continuity of patient care/or patient-centered care/Family Practice/general practitioners/or physicians, family/or physicians, primary care/community health services/or community health nursing/or consumer participation/or counseling/or preventive health services/

(Primary adj1 (care or health)).tw. (family adj1 (doct$ or medic$ or pract$ or physic$)).tw.

#### 2. *Embase:* 224 search results

Health literacy/Literacy:

Exp health literacy/or exp educational status/or exp health education/or exp patient education/health knowledge.mp./exp attitude to health/exp patient compliance/patient attitude/interactive health literacy.tw./critical health literacy.tw./(functional adj health adj literacy).tw.

Population:

Exp cultural deprivation/exp homelessness/vulnerable population/offender/prisoner/lowest income group/poverty/exp social status/refugee/immigrant/asylum seek$.mp./alcoholism/exp drug abuse/exp American Indian/exp Eskimo/exp Aborigine/exp indigenous people/exp socioeconomics/

Risk factors:

Feeding behavior/health behavior/exercise/or “physical activity, capacity and performance”/obesity/or body weight disorder/or overnutrition/overweight.mp./exp body weight/lifestyle/or “lifestyle and related phenomena”/or lifestyle modification/diet/or nutrition/nutritional disorder/or physical disease by body function/or feeding disorder/ or overnutrition/overnutrition/or nutritional disorder/or hyperalimentation/ or obesity/

Primary Health Care:

Exp primary health care/patient care/exp primary medical care/or exp general practice/exp general practitioner/exp primary medical care/community care/exp community health nursing/preventive health service/family medicine/

Intervention for patients:

Health education/or exp health promotion/nutrition education/or exp patient education/exp motivation/motivation$ interview$.mp./exp health literacy/exp medical information/exercise/or “physical activity, capacity and performance”/brief intervention.mp./exp nutritional assessment/self care/self care education.mp./group education.mp./telemedicine/computer assisted therapy/computer assisted information.mp./teacher/patient centered care.mp./patient navigation.mp./coach$.mp./broker$.mp./behavior therapy/exp risk reduction/

Intervention for health care providers:

Facilitation.mp./total quality management/decision support system/health communication.mp./information seeking/exp persuasive communication/

Health education/exp health literacy/or exp patient education/professional education.mp./in service training/personnel management/

#### 3. *Scopus:* 375 search results

Health literacy/Literacy:

“Health literacy” or “patient education” or “health education” or “attitude to health” or “health knowledge” or “health attitude” or “health practice”

Risk factors:

Obesity or weight or body mass index or overnutrition or “life style” or diet or overweight

Primary Health Care:

“Primary health care” or “primary care” or “family practice” or “general practitioner*” or “family physician” or “preventive health service*”

#### 4. *CINAHL:* 179 search results

(MH health education OR MH patient education OR MH health knowledge OR MH attitude to health OR MH patient compliance OR MH literacy OR MH education) AND (MH primary health care OR MH family practice OR MH community health services OR MH community health nursing OR MH patient centered care OR MH continuity of patient care) AND (MH body weight OR MH nutrition disorders OR MH life style OR MH diet OR MH exercise OR MH weight loss OR MH obesity) AND (health education OR patient education OR health knowledge OR attitude to health OR patient compliance OR literacy OR education) AND (primary health care OR family practice OR community health services OR community health nursing OR patient centered care OR continuity of patient care) AND (body weight OR MH nutrition disorders OR life style OR diet OR exercise OR weight loss OR obesity).

#### 5. *APAIS-Healt*h: 318 search results

(“Patient compliance”) OR (“Health behaviour”) OR (“Health practice”) OR (“Health attitude”) OR (“Health knowledge”) OR (“Literacy”) OR (“patient education”) OR (“health education”) OR (“health literacy”) AND (diet) OR (lifestyle) OR (“body weight”) OR (overweight) OR (“physical activity”) OR (exercise) OR (“health behaviour”) OR (“eating behaviour”) OR (BMI) OR (“body mass index”) OR (weight) OR (obesity).

#### 6. *PsycINFO****:*** 185 search results

Risk factors:

Obesity.mp./overweight.mp./overnutrition.mp./diet.mp./exercise.mp./“physical activity”.mp. lifestyle.mp./“life style”.mp./“body weight”.mp./bmi.mp./“body mass index”.mp./“health behaviour”.mp./“health habit”.mp./“eating behaviour”.mp./“feeding behaviour”.mp./“eating habit”.mp./“eating behaviour”.mp.

Primary Health Care:

“Primary health care”.mp./“primary care”.mp./“general practi$”.mp./“family physician$”.mp. “family practice”.mp./“primary care physician$”.mp./“preventive health service$”.mp. “community health service$”.mp./“Community health nursing”.mp./“comprehensive health care”.mp.

Health Literacy/Literacy:

“Health literacy”.mp./“health education”.mp./“patient education”.mp./“patient compliance”.mp./Literacy.mp./“health knowledge”.mp./“health attitude”.mp./“attitude to health”.mp./“health practice”.mp./“health behaviour”.mp./critical health literacy.tw./(functional adj health adj literacy).tw.

#### 7. *Web of Science:* 37 search results

[Topic = (“health habits” or “attitude to health” or “health behaviour” or “health attitude” or “health knowledge” or “educational status” or literacy or “patient compliance” or “patient education” or “health education” or “health literacy”) AND Topic = (”feeding habits“or “eating habits”or “eating behaviour” or “feeding behaviour” or “health behaviour” or “body weight” or “life style” or lifestyle or diet or “over nutrition” or overnutrition or overweight or obesity or “body mass index” or BMI or exercise or “physical activity”)] AND [Topic = (“preventive health services” or “community health nursing” or “community health services” or “primary care physician” or “family physician” or “general practitioner” or “general practice” or “family practice” or “primary care” or “primary health care”)] AND [Topic = (“cultural deprivation” or homelessness or “vulnerable population” or offender or prisoner or “lowest income group” or poverty or “social status” or refugeeor immigrant or “asylum seeker” or alcoholism or “drug abuser” or “American Indian” or “Eskimo” or Aboriginal or “indigenous people” or socioeconomic)].

#### 8. *Australasian Medical Index:* 127 search results

[((AB:“patient compliance”) OR (AB:“health behavior”) OR (AB:“health behaviour”) OR (AB:“health practice”) OR (AB:“health attitude”) OR (AB:“health knowledge”) OR (AB:literacy) OR (AB:“patient education”) OR (AB:“health education”) OR (AB:“health literacy”))] AND [((AB:“nutritional disorders”) OR (AB:“body mass index”) OR (AB:“body weight”) OR (AB:diet) OR (AB:overnutrition) OR (AB:overweight) OR (AB:obesity) OR (AB:“health behaviour”) OR (AB:“physical activity”) OR (AB:exercise) OR (AB:“eating behaviour”) OR (“feeding behaviour”))].

#### 9. *The Cochrane Library:* 61 search results

[“Patient education”:ti,ab,kw or “patient compliance”:ti,ab,kw or “health education”:ti,ab,kw or “health literacy”:ti,ab,kw or “health knowledge” or “health practice” or “attitude to health”] AND [“physical activity” or exercise:ti,ab,kw or “feeding habit” or “health behaviour”:ti,ab,kw or obesity or overweight:ti,ab,kw or “body weight” or “body mass index”:ti,ab,kw or lifestyle or diet:ti,ab,kw].

#### 10. *PAIS International:* 70 search results

[su(“health literacy”) OR su((“health education” OR “patient education”)) OR su((“health knowledge” OR “health practice”)) OR su(“health attitude”)] AND [su(“primary health care”) OR su((“primary care” OR “community health”)) OR su(“preventive health”)].

#### 11. *Joanna Briggs Institute Library:* 25 search results

‘Health literacy’

#### 12. *Google Scholar:* 56 search results

[obesity or overweight or exercise or “physical activity” or “body weight” or “body mass index” or lifestyle or diet] AND [“health literacy” or “health education” or “patient education”]

### Journals

#### 13. *Health Education & Behavior:* 53 search results

‘Health education’ OR ‘Health literacy’

#### 14. *American Journal of Preventive Medicine:* 96 search results

‘Health education’ OR ‘Health literacy’

#### 15. *Preventive Medicine:* 13 search results

‘Health literacy’

Excluded topics on cancer screening

#### 16. *International Journal of Obesity:* 3 search results

‘Health education’

#### 17. Patient Education and Counseling: 389 search results

[(“Patient compliance”) OR (“Health behaviour”) OR (“Health practice”) OR (“Health attitude”) OR (“Health knowledge”) OR (“Literacy”) OR (“patient education”) OR (“health education”) OR (“health literacy”)] AND [(diet) OR (lifestyle) OR (“body weight”) OR (overweight) OR (“physical activity”) OR (exercise) OR (“health behaviour”) OR (“eating behaviour”) OR (BMI) OR (“body mass index”) OR (weight) OR (obesity)].

Excluded topics on cancer screening.
